# Positive end-expiratory pressure (PEEP) level to prevent expiratory flow limitation during cardiac surgery: study protocol for a randomized clinical trial (EFLcore study)

**DOI:** 10.1186/s13063-018-3046-0

**Published:** 2018-11-26

**Authors:** Elena Bignami, Savino Spadaro, Francesco Saglietti, Antonio Di Lullo, Francesca Dalla Corte, Marcello Guarnieri, Giulio de Simone, Ilaria Giambuzzi, Alberto Zangrillo, Carlo Alberto Volta

**Affiliations:** 1 Department of Anesthesia and Intensive Care, IRCCS San Raffaele Scientific Institute, Via Olgettina 60, 20132 Milan, Italy; 2 Department of Morphology, Surgery and Experimental Medicine, Section of Anesthesia and Intensive Care, University of Ferrara, Via Aldo Moro 8, 44121 Ferrara, Italy; 3 Department of Cardiac Surgery, IRCCS San Raffaele Scientific Institute, Via Olgettina 60, 20132 Milan, Italy; 4 Anesthesiology, Critical Care and Pain Medicine Division, Department of Medicine and Surgery, University of Parma, Viale Gramsci 14, 43126 Parma, Italy

**Keywords:** Protective ventilation, Cardiopulmonary bypass, Respiratory failure, Low tidal volume, Continuous positive airway pressure, Postoperative pulmonary complications

## Abstract

**Background:**

Lung dysfunction commonly occurs after cardiopulmonary bypass (CPB). Randomized evidence suggests that the presence of expiratory flow limitation (EFL) in major abdominal surgery is associated with postoperative pulmonary complications. Appropriate lung recruitment and a correctly set positive end-expiratory pressure (PEEP) level may prevent EFL. According to the available data in the literature, an adequate ventilation strategy during cardiac surgery is not provided. The aim of this study is to assess whether a mechanical ventilation strategy based on optimal lung recruitment with a best PEEP before and after CPB and with a continuous positive airway pressure (CPAP) during CPB would reduce the incidence of respiratory complications after cardiac surgery.

**Methods/design:**

This will be a single-center, single-blind, parallel-group, randomized controlled trial. Using a 2-by-2 factorial design, high-risk adult patients undergoing elective cardiac surgery will be randomly assigned to receive either a best PEEP (calculated with a PEEP test) or zero PEEP before and after CPB and CPAP (equal to the best PEEP) or no ventilation (patient disconnected from the circuit) during CPB.

The primary endpoint will be a composite endpoint of the incidence of EFL after the weaning from CPB and postoperative pulmonary complications.

**Discussion:**

This study will help to establish a correct ventilatory strategy before, after, and during CPB. The main purpose is to establish if a ventilation based on a simple and feasible respiratory test may preserve lung function in cardiac surgery.

**Trial registration:**

ClinicalTrials.gov, ID: NCT02633423. Registered on 6 December 2017.

**Electronic supplementary material:**

The online version of this article (10.1186/s13063-018-3046-0) contains supplementary material, which is available to authorized users.

## Background

General anesthesia is associated to a decreased functional residual capacity (FRC) [1]. Many pathophysiological factors, such as high oxygen concentrations delivered at anesthesia induction, supine positioning, muscle relaxation, large amounts of intravenously administered fluids, and also the inflammatory reaction, may be implicated. Due to this phenomenon, the closing capacity may exceed the FRC during general anesthesia, leading to the collapse of the small airways, clinically detectable as expiratory flow limitation (EFL), and atelectasis. Several factors contribute to EFL during general anesthesia, including the clinical characteristics of the patients, anesthesia induction by itself, and also cardiopulmonary bypass (CPB). The presence of tidal EFL implies increased ventilation/perfusion ratio mismatch, increased airways’ resistance, risk of peripheral airways’ injuries, and, histologically, rupture of the alveolar attachments to the respiratory bronchioles, damage of the bronchiolar epithelium and increased number of polymorphonuclear leukocytes in the alveolar walls. The presence of EFL can be easily detected during general anesthesia by using the positive expiratory pressure (PEEP) test. Expiratory flow limitation was detected in a supine position in 10% of the patients during the pre-surgery evaluation. However, this percentage increased to 52% at the end of surgery. In other words, 42% of the patients treated with zero PEEP became flow limited during surgery [1]. EFL is important not only because older patients can be flow limited before surgery, but, more interestingly, because they can develop EFL during surgery.

There are numerous clinical situations associated with the presence of EFL [2, 3], such as heart failure, chronic obstructive pulmonary disease, obesity, sleep apnea, and bronchiectasis. Cardiopulmonary bypass is associated with direct lung damage. Pulmonary atelectasis, apnea, ischemia during CPB and activation of proteolytic enzymes in the pulmonary circulation influence the incidence of postoperative pulmonary dysfunction after cardiac surgery [4–7]. Moreover, although postoperative impairment could be transient and respiratory function can recover shortly after surgery, some patients may develop respiratory complications both in the intraoperative or postoperative period. The incidence of postoperative pulmonary complications (PCCs) ranged from 3 to 16% following coronary artery bypass grafting and 5–7% following cardiac valvular surgery.

The consequence of a low-tidal-volume ventilation strategy can be avoided by the application of PEEP. The use of PEEP prevents the decrease of FRC and can stabilize the small airways and improve respiratory mechanics and oxygenation, provided that PEEP is high enough to prevent the development of EFL. There are several studies that compare high versus low PEEP levels during general anesthesia for major abdominal surgery [1, 8–11], far fewer in adult cardiac surgery. Since the presence of EFL has been previously associated with PPCs after major abdominal surgery [2] and cardiac surgery [12], we hypothesized that the use of PEEP able to avoid EFL should reduce the PPCs during cardiac surgery.

### Objectives

EFL occurs when, at a given lung volume, the expiratory flow is independent of the patient’s expiratory effort or on an increase of elastic recoil pressure [13]. The purpose of this study is to investigate whether an optimal lung recruitment strategy before CPB, based on a best PEEP calculated according to the PEEP test, and during CPB, with a continuous positive airway pressure (CPAP) instead of no ventilation, would reduce the incidence of the EFL phenomenon and of PPCs after cardiac surgery, compared with no PEEP before, during, and after CPB.

The main hypothesis of this trial is that an adequate lung recruitment based on the best PEEP before and after CPB, defined as the minimum PEEP level necessary to avoid the EFL phenomenon, and/or an adequate level of CPAP during could reduce lung injury following cardiac surgery.

## Methods/design

### Trial design

This will be a single-center, single-blind, parallel-group, randomized controlled trial. Using a 2-by-2 factorial design, high-risk adult patients undergoing elective cardiac surgery will be randomly assigned to receive either a best PEEP (calculated with a PEEP test) ventilation or a zero PEEP strategy before and after CPB and CPAP (equal to the best PEEP) or no ventilation (patient disconnected from the circuit) during CPB.

**Fig. 1:**
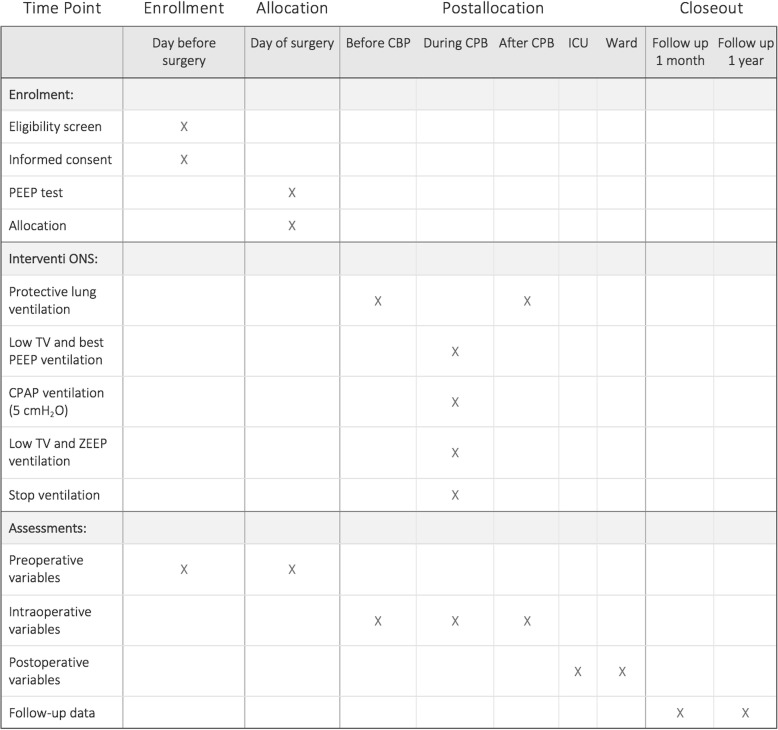
The Standard Protocol Items: Recommendations for Interventional Trials (SPIRIT) Figure of this trial

We will recruit all patients that will match the eligibility criteria at the preoperative evaluation. The protocol structure was written in according to the Consolidated Standards of Reporting Trials (CONSORT) 2010 Statement [14] and follows the Standard Protocol Items: Recommendations for Interventional Trials (SPIRIT) Statement [15]. The SPIRIT Figure of this trial is illustrated in Fig. 1. The SPIRIT Checklist of this trial can be found in Additional file 1. Patients will also be evaluated in terms of (1) shunt fraction and (2) data of respiratory mechanics, such as static compliance, flow, and additional resistance. All these parameters will be determined: (1) immediately after anesthesia induction, (2) before CPB, (3) during CPB, (4) off pump, and (5) end of surgery.

The study has been registered at ClinicalTrials.gov with the registration number NCT02633423 on 6 December 2017.

### Participants

In order to obtain Ethics Committee approval, documentation was submitted in December 2017 and we are currently waiting for approval. We intend to enroll patients aged 18 years or over who are undergoing elective cardiac surgery with planned use of CPB, aortic cross-clamping, median sternotomy, and two-lung ventilation. All patients will provide written informed consent before their inclusion in the trial. The inclusion and exclusion criteria are shown in Table 1.

**Table 1:** Inclusion/exclusion criteria

Eligibility criteria	
Inclusion criteria: • Elective cardiac surgery, with median sternotomy and two-lung ventilation • Patients scheduled for mitral valve regurgitation and/or aortic valve regurgitation surgery, performed with CPB and aortic cross-clamping • Adult patients (age 18 years or older) • Ability to provide informed consent • High risk for respiratory dysfunction, defined as 1 of: preoperative hypoxemia (arterial oxygen saturation < 92% in room air or arterial oxygen partial pressure < 60 mmHg at blood-gas analysis or a PaO_2_/FiO_2_ ratio < 200 at basal blood-gas analysis); preoperative obesity (BMI > 30); preoperative ejection fraction < 50%; preoperative NYHA class > II Age > 65 years [25].	Exclusion criteria: • Non-elective cardiac surgery • Anticipated circulatory arrest, TAVI, Mitraclip • Patient’s refusal • Pregnancy • Thoracotomic approach, with one-lung ventilation • Previous pulmonary resection • Patients with acute kidney injury requiring dialysis • Patients with chronic kidney insufficiency (stage III or greater) • Patients already intubated before arrival in operating theater • Pneumonia in the previous 30 days

*BMI* body mass index, *CPB* cardiopulmonary bypass, *NYHA* New York Heart Association, *TAVI* transcatheter aortic valve implantation

### Endpoints

The primary endpoint will be a composite endpoint of the incidence of EFL after CPB and of PPCs at 5 days after surgery (see Table 2 for complete definition of PPCs). Before/after sternotomy, a PEEP test will provide the best PEEP value, that will be the least value of PEEP for which closing capacity does not exceed the FRC, leading to the avoidance of EFL and atelectasis. In practice, the EFL phenomenon will be assessed at different PEEP levels, until the abrupt decrease in end-expiratory pressure will cause an increment in expiratory flow. At this level the patient will not be flow-limited and this will be considerate his best PEEP for the rest of surgery.

**Table 2:** Definition of postoperative pulmonary complications (PPCs), Jammer et al*.* [24]

Complication	Definition
Respiratory infection	Patient has received antibiotics for a suspected respiratory infection and met one or more of the following criteria: new or changed sputum, new or changed lung opacities, fever, white blood cell count > 12 × 10^9^ l^-1^
Respiratory failure	Postoperative PaO_2_ < 8 kPa (60 mmHg) on room air, a PaO_2_:FIO_2_ ratio < 40 kPa (300 mmHg) or arterial oxyhemoglobin saturation measured with pulse oximetry < 90% and requiring oxygen therapy
Pleural effusion	Chest radiograph demonstrating blunting of the costophrenic angle, loss of sharp silhouette of the ipsilateral hemidiaphragm in upright position, evidence of displacement of adjacent anatomical structures or (in supine position) a hazy opacity in one hemithorax with preserved vascular shadows
Atelectasis	Lung opacification with a shift of the mediastinum, hilum, or hemidiaphragm toward the affected area, and compensatory over-inflation in the adjacent non-atelectatic lung
Pneumothorax	Air in the pleural space with no vascular bed surrounding the visceral pleura
Bronchospasm	Newly detected expiratory wheezing treated with bronchodilators
Aspiration pneumonitis	Acute lung injury after the inhalation of regurgitated gastric contents

*PaO*_*2*_ arterial partial oxygen pressure, *FiO*_*2*_ fraction of inspired oxygen

The secondary endpoints will be the evaluation of the following:

- Readmission to the intensive care unit (ICU)
- Need for re-intubation
- Need for non-invasive ventilation
- Duration of mechanical ventilation
- Postoperative infections
- Major adverse cardiac events (MACE; see Table 3 for complete definition of MACE)
- Length of the ICU and hospital stay
- 30-day and 1-year mortality

**Table 3:** Definition of major adverse cardiac events (MACE), Jammer et al. [24]

Complication	Definition
Non-fatal cardiac arrest	An absence of cardiac rhythm or presence of chaotic rhythm requiring any component of basic or advanced cardiac life support
Acute myocardial infarction	Increase and gradual decrease in troponin level or a faster increase and decrease of creatine kinase isoenzyme as markers of myocardial necrosis in the company of at least one of the following: ischemic symptoms, abnormal Q waves on the ECG, ST-segment elevation or depression; coronary artery intervention (e.g., coronary angioplasty) or a typical decrease in an elevated troponin level detected at its peak after surgery in a patient without a documented alternative explanation for the troponin elevation
Congestive heart failure	New in-hospital signs or symptoms of dyspnea or fatigue, orthopnea, paroxysmal nocturnal dyspnea, increased jugular venous pressure, pulmonary râles on physical examination, cardiomegaly, or pulmonary vascular engorgement
New cardiac arrhythmia	ECG evidence of atrial flutter, atrial fibrillation, or second- or third-degree atrioventricular conduction block
Angina	Dull, diffuse, substernal chest discomfort precipitated by exertion or emotion and relieved by rest or glyceryl trinitrate

*ECG* electrocardiogram

### Interventions (randomization and treatment protocol)

Once the patient has provided informed consent, the investigator (e.g., the anesthesiologist in the operating theater) logs into a dedicated on-line portal (www.eflvent.it) to obtain the allocation arms. The randomization will be in a 1:1 ratio between the parallel groups, in blocks of 20 patients. From that moment, the online data will be anonymous and, in any event, the patient will be analyzed according to the “intention-to-treat” principle.

To reduce the bias, data will be collected by trained observers, trained accordingly to Good Clinical Practice (GCP) guidelines [16, 17], who will not participate to patient care. Furthermore, we decided to focus on clinically relevant data that cannot be influenced by physician management. Caregivers will be interviewed daily about the occurrence of postoperative adverse events. The patients will be blinded to the allocation arm.

All patients will receive lung protective ventilation, with a tidal volume of 7 ml/Kg of ideal body weight (IBW), a respiratory frequency titrated to maintain normocapnia, assessed by seriated blood-gas analyses. FiO_2_ will never be higher than 80%, except for the blood samples obtained for shunt fraction calculation.

Patients will be randomized, immediately before surgery, to receive either a PEEP equal to the best PEEP, assessed with a PEEP test, immediately after the induction of anesthesia or to zero. All patient will be furthermore randomized to receive either a CPAP equal to the best PEEP or a zero PEEP strategy (with disconnection from anesthesia circuit) during CPB. The two randomizations will be independent each from other.

During CPB our goal will be to maintain PaO_2_ between 200 and 250 mmHg in order to avoid hyperoxia-induced lung injury; moreover, the hematocrit will be maintained above 24% [18].

**Fig. 2:**
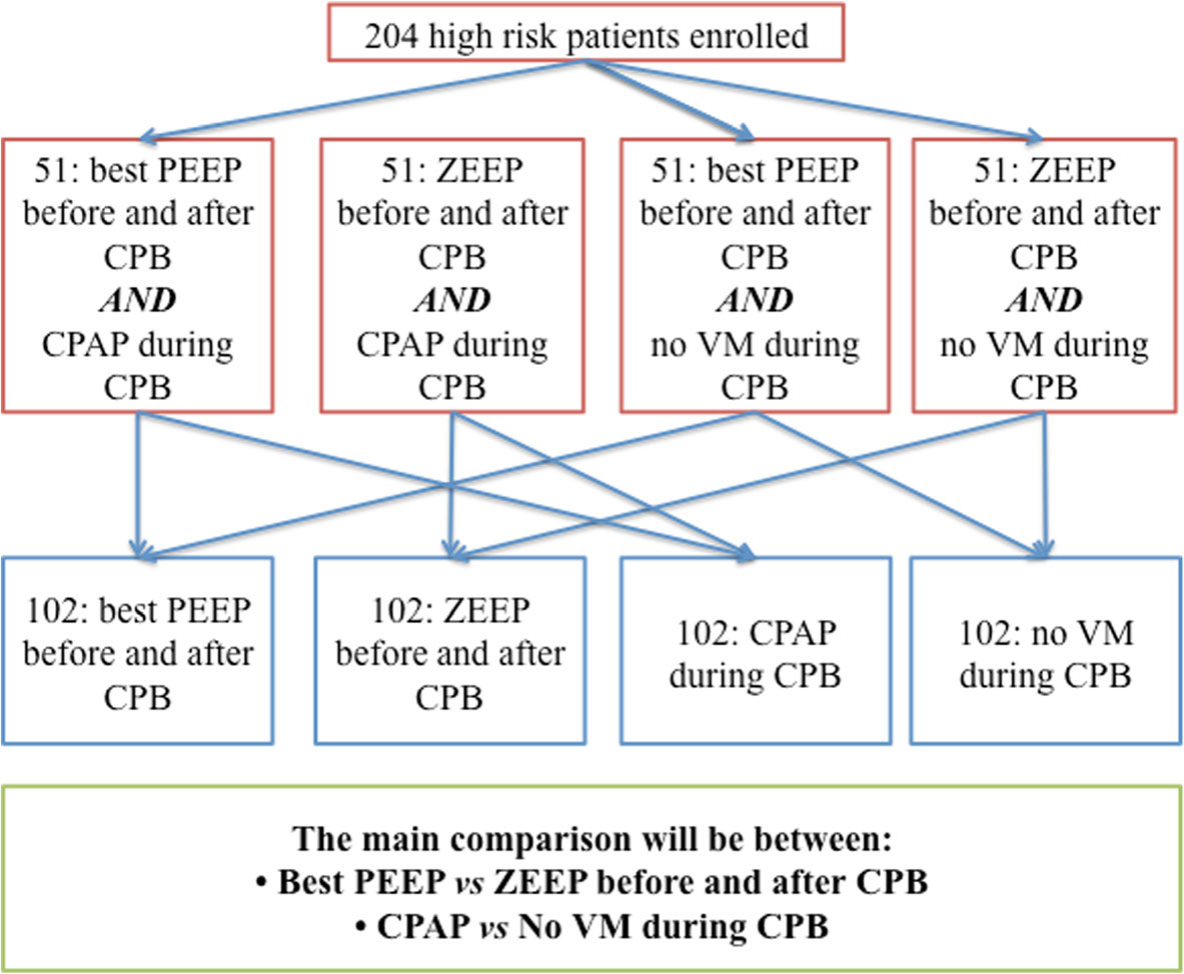
The trial flowchart. Description of the ventilatory strategies and allocation of patients before, during, and after cardiopulmonary bypass

During weaning from CPB we will perform a single alveolar recruitment maneuver (RM). This RM will be performed manually by the anesthesiologist with a gas mixture of oxygen and air (with an inspired oxygen fraction lower than 80%) at the end of procedure. After CPB, this RM will be performed manually, in correspondence to the surgical de-airing procedure. Airway pressure will be kept at 40 cmH_2_O for at least 10 s. The flowchart in figure 2 (Fig. 2) resume the enrollment and allocation process.

### EFL determination

**Fig. 3:**
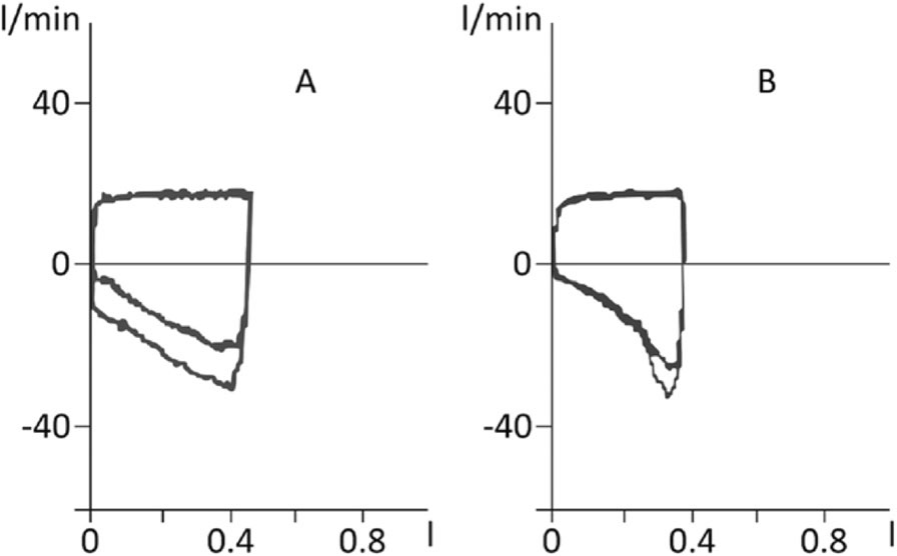
The PEEP test. Flow-volume loops of patients undergoing positive end-expiratory pressure (PEEP) test. **a** Subtraction of 3 cmH_2_O of PEEP in this cohort of patients leads to an increase of expiratory flow: these patients are classified as not flow-limited. **b** Subtraction of 3 cmH_2_O of PEEP does not increase expiratory flow, except for a brief, initial, transient increase, which is mainly the result of a sudden reduction of volume of the upper airways and denotes flow limitation. See text for further explanations

The determination of EFL during general anesthesia and neuromuscular blockade will be performed using the PEEP test. This test is based on a sudden decrease of expiratory resistance obtained by a subtraction of 3 cmH_2_O of PEEP during expiration (Fig. 3). A patient will be considered to have EFL if reducing PEEP by 3 cmH_2_O will not increase expiratory flow when compared with the previous breath (panel B). If expiratory flow will increase with PEEP reduction, the patient will be considered not flow limited (panel A). All loops will be saved in electronic format for further analysis.

EFL will be determined at the following time-points:

- After anesthesia induction
- After sternotomy
- During CPB, immediately before the RM
- After the RM
- Before sternosynthesis
- Before discharge from operating room

After ICU arrival, a best PEEP will be determined in patients having EFL and set for all the postoperative ICU stay. The best PEEP will be defined as the PEEP value able to eliminate EFL, assessed with the same method of the PEEP test. Patients without EFL at arrival in ICU will be ventilated with a PEEP of 5 cmH_2_O. An overview of measurements is provided in Table 4.

**Table 4:** Synopsis of measurements

	Presence of EFL	Lung compliance	Airway resistance	BGA	Shunt percentage
Before sternotomy	X	X	X	X	X
After sternotomy	X	X	X	X	
Before recruitment maneuver	X	X	X		
After recruitment maneuver	X				
Before sternosynthesis	X	X	X	X	
After sternosynthesis	X	X	X	X	X

*BGA* blood-gas analysis, *EFL* expiratory flow limitation

#### Lung mechanics determination [19]

Quasi-static compliance of the respiratory system (Cqst,rs) will be calculated as:

$$ \mathrm{Tidal}\ \mathrm{volume}/\left(\mathrm{End}\ \mathrm{inspiratory}\ \mathrm{plateau}\ \mathrm{pressure}- PEEPtot\ \left(\mathrm{ml}/{\mathrm{cmH}}_2\mathrm{O}\right)\right), $$

where *PEEPtot* is the end-expiratory pressure at period of no flow. Measurement will be performed with an inspiratory pause of 60%.

Dynamic compliance of respiratory system (Cqdyn,rs), induced by thorax opening, will be calculated as:

$$ \mathrm{Tidal}\ \mathrm{volume}/\left(\mathrm{End}\ \mathrm{inspiratory}\ \mathrm{peak}\ \mathrm{pressure}- PEEPtot\ \left(\mathrm{ml}/{\mathrm{cmH}}_2\mathrm{O}\right)\right) $$

Measurement will be performed with an inspiratory pause of 10%.

Airway resistance (Rmin,rs) will be calculated as:

$$ \left( Ppeak-{P}_1\right)/{V}^{'}, $$

where *Ppeak* is the peak inspiratory pressure, *P*_1_ is the airways’ pressure at the point of zero flow and *V*′ is the inspiratory flow. Measurement will be performed with an inspiratory pause of 60%. Lung mechanics will be determinate immediately after every PEEP test execution.

#### Dead space calculation

Dead space fraction calculation will be performed with the Enghoff modification of the Bohr equation:

$$ Vd/ Vt=\left({PaCO}_{2-}\ {PECO}_2\right)/{PaCO}_2, $$

where:

- *Vd* is the dead space
- *Vt* tidal volume
- *PECO*_*2*_ is pressure of mean expired CO_2_

#### Shunt fraction calculation

Shunt fraction will be determined before surgery and after surgery in the operating room during general anesthesia, before patient discharge from operating room. Shunt fraction will be assessed as follows in all patients:

$$ Qs/ Qt=\left({PAO}_2-{PaO}_2\right)\times 0.0031/C\left(a- vmixed\right){O}_2+\left[\left({PAO}_2-{PaO}_2\right)\times 0.0031\right], $$

where:

- *PAO*_*2*_ is oxygen alveolar concentration
- *PaO*_*2*_ is oxygen arterial concentration
- *C(a-vmixed)O*_*2*_ is arteriovenous difference in oxygen concentration
- 0.0031 is a conversion factor to volume percent for O_2_

Measure will be performed while breathing 100% oxygen for 20 min, to achieve a complete hemoglobin saturation.

If a PAC is placed, according to clinical indications, the shunt fraction will be calculated from the following formula:

$$ Qs/ Qt={ CcO 2}_{-}\ {CaO}_2/{CcO}_2- Cv(mixed){O}_2\times 100, $$

where:

- *CcO*_*2*_ is pulmonary capillary blood O_2_ content, estimated with the following equation:

$$ {CcO}_2=\left(\mathrm{Hb}\times 1.34\right)+\left(0.0031\times {PAO}_2\right), $$

- *CaO*_*2*_ is arterial oxygen content
- *Cv(mixed)O*_*2*_ is oxygen content in blood samples obtained from the pulmonary artery
- *PAO*_*2*_ is alveolar oxygen partial pressure

Blood samples will be collected from the arterial catheter and the pulmonary artery catheter, under a FiO_2_ of 100% in the same moment.

Furthermore, blood-gas analyses (BGA) will be performed on arterial blood:

- After anesthesia induction
- After sternotomy, together with heparinization
- Before sternosynthesis, together with protamine administration
- Before discharge from the operating room

### Postoperative ventilation

Mechanical ventilation setting used during patient transfer from the operating theater to the ICU will be reported in appropriate case report form (CRF). In the ICU it will be applied a volume-controlled continuous mandatory ventilation (VC-CMV) with the same parameters used in the operating room. Blood oxygen saturation will be constantly monitored with a pulse oximeter; blood-gas analyses will be performed according to clinical needs.

Following data will be collected: extubation time, duration of mechanical ventilation and need for re-intubation.

### Intraoperative monitoring

Intraoperative monitoring will include: electrocardiogram (ECG), pulse oximeter, capnography, urine output, invasive blood pressure measurements, advanced hemodynamic monitoring (pulmonary artery catheter (PAC)/transesophageal echocardiogram (TEE)), bladder or esophageal temperature, and activated clotting time (ACT).

The anticoagulant protocol in our center is as follows: heparin (3 mg/Kg) to achieve an ACT of 200 s for cannulation and 480 s to proceed with the CPB. At the end of the CPB, protamine (3 mg/Kg) is used (ACT target < 150). In case of allergy to heparin, we will administer bivalirudin.

During CPB, pump flow of 2.5 L/min/m^2^ and mild hypothermia (31–33 °C) will be applied.

The following ventilatory parameters will be monitored: TV, PEEP, FiO_2_, peak airway pressure (Paw), and plateau pressure (Pplat).

### Data collection

All the data will be collected on the dedicated CRF. No personal information of the patients will be included in the database. All patients will receive a code at the moment of randomization and it will be used for the storage in the online database. Data will be stored pseudo-anonymously and only the investigators will be able to match patients’ names and the codes.

Data collection will include:

- Preoperative information: anamnesis, physical examination, cardiac and pulmonary function, laboratory analysis
- Intraoperative data: ventilatory parameters, type of anesthesia, type of cardioplegia, type of CPB circuit, temperature during CPB, use of volatile anesthetics in CPB, volume and type of fluids administered, transfusion requirements, use of vasoactive drugs, duration of intervention, ventilation mode used during transport to the ICU
- Postoperative data: use of inotropic or vasoactive drugs, mechanical devices, time to extubation, need for respiratory support or re-intubation, and hospital stay

In addition, the standard cardiac surgery risk scores, Euroscore I–II [20, 21], the ACEF score [22] and the ARISCAT score [23], will be calculated.

Regular backups will be also done in order to minimize the risk of data corruption.

### Follow-up variables

Follow-up will be performed at 30 days and at 1 year. After discharge from the hospital, patients will be phoned for the follow-up. Any readmission to hospital or Exitus will be recorded as well as information related to major pulmonary complications.

### Statistical considerations

#### Sample size

Sample size calculation was based on a two-sided α error of 0.05 and a 80% power (β). On the basis of our experience, we anticipate that the 50% of patients with ventilation cessation during CPB and no PEEP before and after CPB will reach the composite endpoint of EFL after CPB and respiratory complications at 5 days, while only the 30% of the patients treated with an optimal ventilatory strategy will develop this composite endpoint. Including a drop-out fraction of 10%, we will enroll 51 patients per group, 204 in total.

#### Data analysis

A statistical consultant dedicated, but not involved in patient management, will provide independent consultancy for data-quality checking and statistical analysis, and will be responsible for the statistical analysis. Data will be analyzed by means of SPSS 25.0 software. All data analyses will be carried out according to a pre-established intention-to-treat analysis plan. In addition, per-protocol analyses will be carried out, but only as a means to explore safety and feasibility. We will analyze patients in the treatment group to which they are allocated. Two-sided significance tests will be used. Dichotomous variables will be analyzed using the two-tailed ***χ***^2^ test, using the Yates’ correction when appropriate. Continuous variables will be compared by analysis of variance or the non-parametric Kruskal-Wallis test. Relative risks with 95% confidence intervals and differences between medians with 95% confidence intervals (using the Hodges-Lehmann estimation) will be calculated when appropriate.

The principal comparison will be between the patients who will or will not receive best PEEP ventilation and who will or will not receive CPAP during CPB.

### Trial organization

The IRCCS San Raffaele Scientific Institute is the center for this study. It is responsible for the organization of the trial, development of the randomization scheme, study database, data consistency checks and analysis.

We will create an on-line platform (http://www.eflvent.it) where investigators can electronically randomize every patient and load data in the online CRF. All data will be collected anonymously in the on-line database.

Definitions and use of outcome measures are in accordance with the standards for European Perioperative Clinical Outcome (EPCO) definitions [24].

Safety monitoring activities will be performed by an independent monitoring body, with known experience in the field, and will include reviewing the protocol, data integrity, participant risk and safety; in particular, monitoring adverse events, and data confidentiality. The monitoring body will be separate and independent from the clinical staff or anyone responsible for patient care. The monitoring body will not have scientific, financial, or other conflict of interest related to the trial. Current or past collaborators or associates of the principal investigator will not be a part of the monitoring body. The monitors will follow the Declaration of Helsinki and GCP guidelines. Clinical monitors will verify adherence to required clinical trial procedures and confirm accurate collection of data. Study monitoring and follow-up, from the initial set-up to final reporting, are all fulfilled according to current national and international requirements.

## Discussion

Postoperative lung complications are common after cardiac surgery; when a severe complication does occur, a patient’s life may be significantly threatened.

Many factors can contribute to their development. Among these, the phenomenon of the expiratory flow limitation (EFL) has not been studied in the literature, especially in elective cardiac surgery. This phenomenon seems to correlate with PPCs in other major surgical fields and it could be easily prevented by the choice of an adequate PEEP level. Our commitment to this study has focused particularly on investigating whether optimal lung recruitment before, during and after CPB, associated with protective lung ventilation, would reduce the lung damage in cardiac surgery. The factorial trial design we decided to apply is aimed at distinguishing between what happens during CPB (e.g., lung collapse if no CPAP is applied and inflammatory damage) and before and after CPB (e.g., derecruitment and atelectrauma, without an adequate PEEP level).

### Feasibility and safety of EFL determination

The determination of EFL during general anesthesia and paralysis will be performed using the PEEP test. This test is easy to do and does not require any invasive maneuvers. These studies do not involve additional risks for subjects to whom are offered the best clinical care conditions.

### Limitations

Firstly, the EFLcore trial is a single-center study. This could be a limitation because we may not be adequately representative of a population typical of the patients undergoing cardiac surgery all over the world. It will enroll patients undergoing elective cardiac surgery with CPB, with strict inclusion criteria. We are aware that results obtained from eligible patients that usually have no significant risk factors for postoperative respiratory insufficiency may not apply for a general population; in particular, as a result of restrictions in the inclusion criteria.

Other limitations of this trial could be the accuracy of measurements related to EFL determination. These will be performed while breathing 100% oxygen for no longer than 20 min in order to avoid hyperoxia induced damage. Additionally, a single recruitment maneuver will be performed manually, in correspondence to the surgical de-airing procedure in order to avoid significantly impairing hemodynamic stability during CPB.

Certainly, more studies will be needed to ensure the evidence of results on larger populations of patients undergoing cardiac surgery; however, since this is the first trial to evaluate different ventilation strategies during CPB related to the EFL phenomenon, we thought it appropriate to create a clear experimental setting.

### Current trial status

The documentation was submitted to the San Raffaele Hospital Ethics Committee in December 2017 and we are currently waiting for approval before starting recruitment. Patient recruitment will begin in September 2018. The final results will be published as soon as the analysis is completed. We estimate 1 year of enrollment to complete the trial. Including 1 year of follow-up, the final data will probably be available in 2020.

## Additional file

Additional file 1: Standard Protocol Items: Recommendations for Interventional Trials (SPIRIT) Checklist. (DOC 123 kb)

## Abbreviations

ACT: Activated clotting time
BGA: Blood-gas analysis
CPAP: Continuous positive airway pressure
CPB: Cardiopulmonary bypass
Cqst,rs: Quasi-static compliance of the respiratory system
EFL: Expiratory flow limitation
EPCO: European Perioperative Clinical Outcome
FRC: Functional residual capacity
GCP: Good Clinical Practice
IBW: Ideal body weight
ICU: Intensive care unit
PAC: Pulmonary artery catheter
PEEP: Positive end-expiratory pressure
PPCs: Postoperative pulmonary complications
Qs: Pulmonary physiologic shunt
Qt: Total cardiac output
RM: Recruitment maneuver
Rmin,rs: Airway resistance
TEE: Trans-esophageal echocardiography
VC-CMV: Volume-controlled continuous mandatory ventilation
Vd: Dead space
Vt: Tidal volume
